# From reductionism to complex systems: Paradigm shift and knowledge evolution in competitive sports research using BERTopic

**DOI:** 10.1371/journal.pone.0346022

**Published:** 2026-03-30

**Authors:** Ling Pan

**Affiliations:** School of Physical Education, Shanghai University of Sport, Shanghai, China; SPRINT - Sport Physical Activity and Health Research & Innovation Center, PORTUGAL

## Abstract

**Background and purpose:**

With the exponential growth in the volume of competitive sports research, traditional bibliometric methods based on keyword co-occurrence struggle to accurately capture the field's deep semantic structure and evolutionary dynamics. This study aims to employ natural language processing (NLP) techniques to reconstruct the knowledge structure and evolutionary characteristics of competitive sports research from a micro-semantic perspective.

**Methods:**

Utilizing the BERTopic topic modeling algorithm combined with dynamic regression analysis, we performed unstructured text mining on 24,659 core abstracts published between 2010 and 2024. Data were retrieved from the Web of Science, PubMed, and Scopus databases.

**Results:**

The study reveals a distinct “monocentric, multi-dimensional” hierarchical structure in competitive sports research.Topic 0 (“Sports Injury and Load Management”) represents the largest volume and exhibits continuous, significant growth (“Hot”). It constitutes the field's epistemological foundation, demonstrating robust vitality driven by technological iteration. Meanwhile, the explosive growth of Topic 1 (“Sports Mental Health”) marks a profound paradigm shift from singular “physiological optimization” to “holistic psychophysical well-being."In contrast, Topic 4 (“Mega-event Management”) shows statistically non-significant growth, indicating that this sub-field has reached a stage of theoretical saturation. Furthermore, the evolution of the discipline can be categorized into three stages: the Budding Period (2010–2014), the Rapid Development Period (2015–2019), and the Multi-dimensional Transition Period (2020–2024).

**Conclusion:**

Competitive sports science is currently in a critical transition period from reductionism to a complex systems perspective. Future research frontiers are likely to focus on the deep integration of biomedical technologies and humanistic psychology. This study provides a novel methodological perspective for understanding the evolutionary laws of the discipline.

## 1. Introduction

Competitive sports is fundamentally defined as a systematic pursuit aimed at maximizing human potential—spanning physical, psychological, and athletic capabilities—to achieve peak performance via scientific training and competition [1]. As a distinct pillar of modern sports, it is differentiated from school or recreational sports by its inherent high-stakes competitiveness and goal-oriented nature [2]. Structurally, competitive sports operates as a complex ecosystem comprising four interconnected dimensions: human agents (athletes and coaches), the training process, the competitive event, and the external material infrastructure (facilities, equipment, and funding) [3]. This multi-faceted composition has been corroborated by scholars such as Sottas [4] and Hardy [5], who emphasize that the operational morphology of competitive sports relies on the synergy between human actors and material support.

Driven by advancements in foundational sports sciences, research in competitive sports has increasingly trended towards diversification, technological integration, and systematization [6]. Notably, the global dominance of developed Western nations—in both athletic performance and scientific inquiry—has fostered a distinct interdisciplinary landscape. Consequently, research themes have expanded beyond physiological metrics to encompass socio-cultural dimensions such as race [7,8], gender [9–11], and migration [12,13].However, this rapid proliferation of literature has also unveiled critical bottlenecks: the field is approaching theoretical saturation, research cycles are elongating, and there is a marked scarcity of comparative studies across different sports disciplines, geographical regions, and nations [14]. A pressing challenge remains the translation of academic findings into practical applications [15]. Bridging this “theory-practice gap” is essential for addressing the persistent conundrums that have long plagued the competitive sports sector [16].Despite these challenges, existing literature reviews often lack a comprehensive framework to accurately trace the evolutionary trajectory or scientifically forecast future trends. Consequently, a systematic and data-driven analysis of the knowledge evolution in competitive sports research is imperatively needed.

Tracing the evolutionary dynamics of specific research themes necessitates the processing of massive datasets. Traditional systematic reviews, however, rely heavily on manual coding, a process that is not only labor-intensive but also susceptible to subjective bias and limited scalability.While computational approaches have been introduced to address these limitations, conventional topic modeling algorithms—such as Latent Dirichlet Allocation (LDA) [17], Probabilistic Latent Semantic Analysis (PLSA), and Non-negative Matrix Factorization (NMF) [18]—predominantly rely on the Bag-of-Words (BoW) assumption. Consequently, these models often disregard word order and fail to capture the deep, contextual semantics embedded within the text, leading to simplified segmentation and less accurate topic representation.In contrast, BERTopic emerges as a state-of-the-art solution. By leveraging transformer-based language models (specifically BERT) to generate contextualized word embeddings, BERTopic transcends the limitations of BoW approaches. It effectively captures the nuanced semantic relationships between words, resulting in more precise and scientifically robust sentence representations. Furthermore, BERTopic integrates dimensionality reduction and clustering to visualize inter-topic correlations and their evolutionary trajectories, offering a robust framework for probabilistic topic assignment [19].A distinguishing feature of BERTopic is its unsupervised learning architecture. This minimizes human intervention during the data processing stage, thereby significantly enhancing the objectivity and reproducibility of the findings. To date, this methodology has been successfully applied across diverse disciplines to map complex knowledge landscapes [20,21].

As of 2024, the cumulative volume of global academic literature on competitive sports has exceeded 20,000 articles. The magnitude of this scholarly output has rendered it well beyond the processing capacity of traditional manual systematic reviews. Consequently, the synthesis of competitive sports research has entered a critical new phase, creating an imperative for more efficient and precise analytical paradigms to navigate this information overload.To address this challenge, this study employs the machine learning-based BERTopic framework. Adopting a topic clustering approach, we aim to:Deconstruct the evolutionary dynamics of distinct research themes within the field;Elucidate the intrinsic interconnections and developmental trajectories among these topics;Evaluate their academic impact across different periods and unveil their phasic characteristics within shifting historical contexts.Beyond merely synthesizing fragmented data, this study provides an objective forecast of emerging research frontiers, offering robust data-driven scaffolding for the future development of competitive sports science.

## 2. Literature review

### 2.1 Disciplinary dynamics and evolutionary patterns of competitive sports research

In recent years, the disciplinary attributes of competitive sports research have shifted from a monodisciplinary focus to a diversified landscape, evolving into a complex, multidimensional domain. This evolution is characterized by the cross-fertilization of fields such as exercise physiology, biomechanics, psychology, nutrition, sports medicine, management science, and engineering.For instance, regarding fatigue management in elite athletes, Robson [22] posits that effective strategies must transcend the traditional boundaries of sports science. He advocates for a comprehensive approach that encompasses self-assessment, data monitoring, and the adjustment of pedagogical methods. Similarly, in the context of fatigue monitoring, Li [23] bridged the gap between management science and artificial intelligence by integrating fuzzy decision support theory with AI algorithms to develop a real-time monitoring system for athletes.Concurrently, driven by the rapid acceleration of digital technologies, the research landscape is being significantly propelled by emerging frontiers such as wearable devices, AI applications, and electronic sports (e-sports). This technological imperative has been corroborated by recent empirical studies from Koshy [24], Smerdov [25], and Yang [26].

Evidently, this dynamic interdisciplinary evolution has resulted in the dispersion of competitive sports literature across a heterogeneous array of journals and databases, fostering a highly fragmented and semantically complex knowledge ecosystem.Existing methodological approaches face significant limitations in navigating this complexity:Traditional narrative reviews, which rely heavily on manual induction by domain experts, increasingly struggle to capture the field's holistic knowledge architecture and intrinsic associations with objectivity and comprehensiveness.Systematic reviews and meta-analyses, while maintaining methodological rigor, are typically confined to pre-defined, narrow inquiries—such as evaluating the efficacy of specific interventions on isolated metrics—thereby lacking a macro-level perspective.Bibliometric tools like CiteSpace excel in mapping publication volumes and collaboration networks but fail to penetrate the deep semantic themes hidden within the text or track their subtle evolutionary nuances.Consequently, to achieve an objective, dynamic, and scalable resolution of the knowledge structure in competitive sports, there is an urgent need to incorporate Computational Social Science approaches. The adoption of automated text mining and topic modeling technologies represents not merely an option, but an inevitable methodological trend for future advancement.

### 2.2 Topic modeling in sports science: Current applications and methodological bottlenecks

Early-stage sports science research predominately relied on traditional bibliometric indicators (e.g., publication counts, citation analysis, H-index) and simple co-word analysis to dissect research trends. However, with the exponential growth of computational power, topic modeling—as an unsupervised machine learning technique—has gained prominence for its ability to automatically uncover latent themes within textual data. Over the past decade, Latent Dirichlet Allocation (LDA) and its variants have established themselves as the dominant paradigm in this domain.For instance, Na et al.[27] employed LDA to analyze massive sports news datasets to construct a metaverse database;Lee and Han [28] leveraged it to explore psychological dimensions in football research, laying a foundation for subsequent inquiries;Hoang et al. [29] extended its application to predict outcomes in baseball matches. In essence, LDA facilitated a significant methodological leap from rudimentary “word frequency statistics” to sophisticated “topic probability distributions,” providing a foundational framework for summarizing the status quo and exploring future trends.

Notwithstanding its contributions, LDA exhibits inherent limitations when addressing the semantic complexity of modern competitive sports research:The “Bag-of-Words” Constraint: LDA fundamentally treats documents as an unordered “Bag-of-Words" (BoW), completely disregarding sequential word order and contextual nuances. Consequently, it struggles with polysemy disambiguation. For example, it fails to precisely distinguish the semantic difference of the word “load” between “training load monitoring” (physiological) and “cognitive load” (psychological).Limited Interpretability: Topics generated by LDA often manifest as disjointed lists of high-frequency terms (e.g., “athlete,” “protein,” “anxiety”) that lack intrinsic logical coherence. This necessitates substantial subjective interpretation by researchers to make sense of the clusters, thereby reducing the model's direct usability and objectivity.Static Nature: LDA is inherently a static model. While it can simulate evolution through “time-slicing,” it fails to objectively capture the continuous, dynamic evolutionary trajectory of research themes over time.

### 2.3 Methodological advancement: The BERTopic framework for deep semantic and dynamic evolutionary modeling

Built upon the robust architecture of pre-trained language models, BERTopic excels in comprehensively capturing the contextual semantics, structural nuances, and sequential dependencies of text [30]. Particularly when processing short-text data (such as abstracts), it employs advanced dimensionality reduction and density-based clustering algorithms (e.g., UMAP and HDBSCAN) to objectively filter noise and outliers, demonstrating exceptional robustness [31]. Consequently, the topics generated possess superior interpretability. By integrating Class-based TF-IDF (c-TF-IDF) and Maximal Marginal Relevance (MMR), the extracted topic keywords are rendered highly representative and distinctive. The versatility of BERTopic is evidenced by its widespread application across diverse disciplines: Madrid [32] utilized it to map the evolutionary trajectory of rheumatology; Yang et al. [33] applied it to social media marketing analysis; and Söderwall [34] conducted a comparative study between BERTopic and LDA on policy texts. Collectively, these studies highlight BERTopic's proficiency in evolutionary analysis. By calculating the similarity of topic vectors across time slices, it visualizes shifts in topic intensity, demonstrating a dynamic tracking capability that far surpasses static models.

For the specific context of competitive sports research, BERTopic's deep semantic embedding capability provides a critical advantage in deciphering the context-specific meanings of polysemous terms within multidisciplinary texts. Furthermore, addressing the field's rapid paradigm shifts and high susceptibility to subjective interpretation, BERTopic's non-parametric nature and dynamic analysis features significantly minimize human intervention, thereby ensuring the reproducibility and objectivity of the results. However, despite its proven value across various disciplines, there remains a paucity of research that systematically applies this framework to mine knowledge and trace evolutionary trends in competitive sports literature on a global scale and across extensive longitudinal spans.

### 2.4. Research gap

A synthesis of prior literature reveals that current reviews in competitive sports predominantly rely on manual induction or conventional machine learning techniques. There is a notable absence of large-scale, dynamic knowledge discovery utilizing deep learning-based, context-aware topic models. Consequently, existing approaches suffer from significant limitations regarding accuracy and processing efficiency. Furthermore, the field has yet to fully leverage advanced tools like BERTopic—which possesses the capacity to autonomously determine topic counts, decipher professional semantics, and visualize evolutionary paths—resulting in a perceptible methodological lag in uncovering the deep-seated, dynamic knowledge architecture of the discipline.

To bridge these gaps, this study systematically integrates the BERTopic framework into the domain of competitive sports research. The primary objective is to generate empirical insights regarding the evolutionary landscape, current research frontiers, potential interdisciplinary hotspots, and future trends. These findings aim to provide researchers with objective, quantitative, and prospective decision support for navigating domain dynamics.Specifically, this study addresses the following four Research Questions:

RQ1: What is the typological structure of research topics within the field of competitive sports?

RQ2: Which topics occupy a dominant position within the knowledge network?

RQ3: What are the developmental characteristics and phasic shifts across different temporal periods?

RQ4: What are the emerging trends and potential future directions for the field?

## 3. Methodology

### 3.1. Research framework

**Fig 1:**
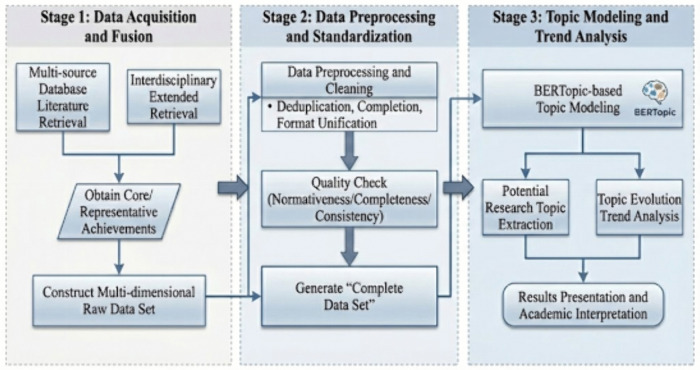
The methodological framework of competitive sports research based on the BERTopic model.

As illustrated in Fig 1, the methodological workflow of this study is structured into some interconnected phases:Initially, a comprehensive literature retrieval is conducted across multiple authoritative databases to acquire the most influential and representative scholarly outputs related to competitive sports;Through a broad, cross-disciplinary search strategy, the study captures diverse research findings from varying fields, thereby constructing a rich, multi-dimensional raw dataset;The raw data undergoes rigorous preprocessing (e.g., de-duplication, cleaning). The resulting dataset, designated as the “Finalized Dataset” (or Corpus), ensures data integrity and consistency, laying a solid foundation for subsequent analysis;Finally, the BERTopic model is deployed on the “Finalized Dataset” to extract latent research themes and analyze their evolutionary trends over time.

### 3.2. Data acquisition and preprocessing

A systematic search strategy was implemented to construct the corpus. The search was conducted within the Abstract fields of four major international databases: Web of Science (Core Collection), Scopus, PubMed, and ScienceDirect. The temporal scope was set from January 1, 2010, to December 31, 2024. The specific boolean search query was constructed as follows:TS = (“competitive sport*” OR “elite sport*” OR “high performance sport*” OR “professional sport*” OR “olympic*” OR “paralympic*” OR “championship*” OR “elite athlet*” OR “competitive athlet*”).The initial retrieval yielded 26,196 records. Subsequently, strict inclusion and exclusion criteria were applied: Only peer-reviewed journal articles were included;Exclusions: Retracted publications, anonymous works, and records with missing abstracts were removed. Following a rigorous manual screening process based on these criteria, a raw dataset of 25,121 articles was established.To ensure data quality, preprocessing was performed using Python 3.11.4(see S1 Data for the raw dataset). We utilized the NLTK library to execute the following cleaning procedures:Converting all text to lowercase and splitting text into tokens;Eliminating standard English stopwords (e.g., “the,” “is”) and domain-specific generic academic terms (e.g., “study,” “result,” “method”) that contribute little semantic value. After cleaning, the final corpus comprised 24,659 valid articles. The finalized dataset was structured in CSV format containing two primary columns: “Abstract” (textual data) and “Year” (temporal metadata).

### 3.3. BERTopic modeling implementation

**Fig 2:**
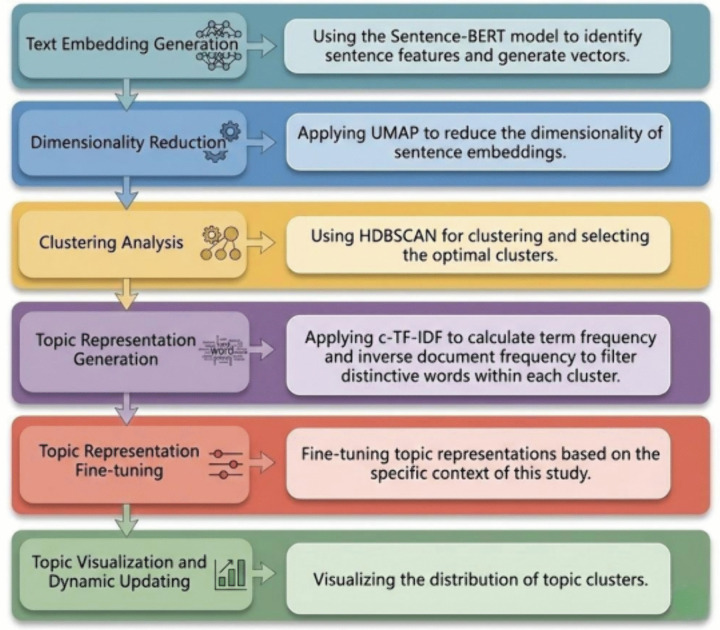
Schematic representation of the BERTopic modeling pipeline.

As shown in Fig 2, it encompasses all stages of the bertopic model's operation.

#### 3.3.1. Document embedding generation.

BERTopic leverages pre-trained language models to generate embeddings for the corpus. These embedding models are designed to capture deep semantic information and contextual dependencies, mapping each textual unit (whether a sentence or a paragraph) into a high-dimensional vector representation. This embedding mechanism is critical as it not only preserves the intrinsic semantic features of the text but also establishes a robust foundation for subsequent clustering and topic extraction phases.Specifically, architectures like Sentence-BERT (SBERT) enhance vector expressiveness by incorporating context-dependent properties, rendering them highly effective for complex topic modeling tasks [35]. By ensuring that semantically similar texts are mapped to proximal points within the vector space, these embeddings achieve high semantic consistency and representational fidelity.In this study, we selected the “all-mpnet-base-v2” pre-trained model for embedding generation. This model was chosen for its superior performance on semantic search benchmarks, allowing for a more precise identification and representation of the nuanced scientific literature analyzed in this research.

#### 3.3.2. Dimensionality reduction via UMAP.

Given the high dimensionality of the generated embeddings, direct clustering can be prone to the “curse of dimensionality.” Therefore, we employed Uniform Manifold Approximation and Projection (UMAP) to project the sentence embeddings into a lower-dimensional manifold. As a non-linear dimensionality reduction technique, UMAP excels at effectively preserving both the local neighborhood structure and the global structural layout of the data [36].To optimize the embeddings for the subsequent clustering phase, specific hyperparameters were configured as follows:n_neighbors: Set to 15. This parameter controls the size of the local neighborhood considered for each data point, balancing the preservation of local logic against global structure.n_components: Set to 5. Following established precedents in topic modeling literature, reducing embeddings to 5 dimensions retains sufficient semantic information while significantly reducing computational complexity.Distance Metric: Cosine similarity was utilized to measure the distance between data points, which is standard for high-dimensional text embeddings.min_dist: Set to 0.0. Unlike visualization tasks (where a higher value prevents overlap), setting the minimum distance to 0.0 allows embeddings to cluster tightly, which is advantageous for the subsequent density-based clustering algorithm.

#### 3.3.3. Clustering via HDBSCAN.

Following dimensionality reduction, we employed HDBSCAN (Hierarchical Density-Based Spatial Clustering of Applications with Noise) to identify dense clusters of document vectors. Unlike partition-based methods (e.g., K-Means), HDBSCAN is a density-based algorithm that automatically determines the number of clusters and explicitly identifies outliers (noise points) that do not belong to any specific topic. It constructs a hierarchical tree of cluster stability to extract the most persistent structures.To adapt the algorithm to the specific scale and granularity of our dataset, the hyperparameters were configured as follows:min_cluster_size: Set to 60. While the default value is 5, previous studies suggest a minimum of 20 for standard datasets. Given the extensive volume of our corpus (>24,000 articles), a small threshold would result in excessive topic fragmentation (micro-topics). Increasing this value to 60 ensures that only substantial, theoretically meaningful themes are retained.min_samples: Set to 10. This parameter controls the local neighborhood density required for a point to be considered a “core point.” This setting balances the sensitivity of cluster formation with the effective filtration of noise.metric: Set to ‘euclidean.’ This ensures valid spatial measurement within the reduced low-dimensional manifold generated by UMAP.nr_topics = “auto”: We enabled the automatic topic reduction feature. This function merges highly similar topics post-clustering based on their semantic proximity, ensuring the compactness and distinctiveness of the final topic list.These parameter configurations align with best practices in computational linguistics and bibliometrics, providing a tailored approach for analyzing the heterogeneous landscape of competitive sports research.

#### 3.3.4. Topic representation via c-TF-IDF.

Once the clusters are established, the final computational step is to derive semantically meaningful labels for each topic. To achieve this, we employed Class-based TF-IDF (c-TF-IDF), a specialized variation of the traditional TF-IDF algorithm optimized for topic modeling [37].Unlike standard TF-IDF, which operates at the document level, c-TF-IDF treats all documents within a single cluster as a concatenated “meta-document.” The algorithm then calculates the importance of words based on their frequency within this specific class relative to their frequency across the entire corpus. The mathematical intuition is to assign higher weights to terms that are highly frequent within a specific cluster but rare in others.This weighting mechanism effectively highlights words with high discriminative power, filtering out generic terms and ensuring that the generated keyword lists are highly representative and specific to the semantic core of each topic.

#### 3.3.5. Visualization and dynamic evolutionary analysis.

To facilitate intuitive interpretation of the semantic landscape, we utilized UMAP to project the high-dimensional topic distributions into a low-dimensional space (2D or 3D). This generates an Intertopic Distance Map, where the spatial proximity between points reflects the degree of semantic similarity between topics.Building upon this spatial projection, we computed the cosine similarity matrix between topics to perform hierarchical clustering. This analytical step enables the aggregation of granular micro-topics into broader macro-themes (core research directions), providing a structural overview of the field.Furthermore, by integrating temporal metadata (timestamps), we employed the Dynamic Topic Modeling (DTM) module. To quantify and visualize the evolutionary trends, the statsmodels library was utilized to model the fluctuation of topic frequency over time. This approach allows for a rigorous exploration of the developmental trajectories, revealing how specific research foci in competitive sports have waxed or waned across the studied period.

To ensure the reproducibility of our thematic evolution analysis, the Python scripts used for BERTopic modeling—including data preprocessing, embedding generation, and dynamic topic modeling—are provided as S2 Code. These scripts detail the specific hyperparameter settings and the logical flow of our computational analysis.

## 4. Results

### 4.1. Identification of research topics in competitive sports

The BERTopic modeling process extracted a total of 44 distinct research topics (labeled Topic 0 to Topic 43) from the corpus of 24,659 abstracts. Table 1 presents a descriptive summary of selected major topics, detailing their representative keywords (top terms) and the corresponding publication volume (count) for each category.In terms of overall distribution, the results reveal a significant variance in academic focus:Topic 0 (“Sports Injury and Load Management”) exhibits the highest prevalence with the largest number of publications, indicating it constitutes the predominant focus of scholarly attention within the field.This is immediately followed by Topic 1 (“Sports Mental Health”), which ranks second in publication volume, highlighting its growing importance.Conversely, Topic 15 (“Paralympics and Specific Techniques”) comprises the smallest proportion of the dataset. This suggests that, compared to mainstream able-bodied sports, research concerning the Paralympic Games and related technical disciplines currently remains a relatively niche area and has received comparatively limited attention from the academic community.The BERTopic model identified a series of distinct thematic clusters within the competitive sports literature. The comprehensive list of these topic labels and their hierarchical naming is detailed in S3 Table. Furthermore, to provide a granular view of the thematic content, the top representative keywords for each identified topic are provided in S4 Table.

**Table 1:** Overview of representative research topics in the field of competitive sports.

Topic	Count	Representative Words
0	6333	injury,injuries,knee,players,load,soccer,strength,match,return,shoulder
1	1462	mental,health,anxiety,coach,coping,leadership,coaches,coaching,self,cohesion
2	894	concussion,symptom,baseline,head,symptoms,assessment,history,cognitive,memory,impact
3	788	swimmers,race,swimming,pacing,stroke,skiers,rowing,races,ski,skiing
4	723	event,mega,olympic,host,olympics,events,residents,legacy,games,china
5	573	judo,combat,taekwondo,karate,judokas,martial,boxers,wrestlers,boxing,martial arts
6	457	visual,task,cognitive,motor,skilled,eye,brain,ball,expertise,gaze
7	436	home,attendance,leagues,demand,advantage,revenue,league,uncertainty,market,teams
8	395	women,gender,gendered,media,coverage,trans,equality,female, bodies,woman
9	371	teachers,education,teaching,pe,teacher,students,learning,pe teachers,curriculum,pedagogical
10	338	doping,anti,use,supplements,drugs,drug,supplement,alcohol,moral,ds
11	323	disability,disabilities,intellectual,disabled,paralympic,id,’special,people,inclusion,attitudes
12	269	career,transition,retirement,identity,athletic identity,dual,transitions,careers,student,retired
13	256	sleep,sleep quality,quality,night,travel,duration,hygiene,quantity,poor,efficiency
14	242	hydration,fluid,glucose,intake,energy,triad,urine,drink,energy availability,availability
15	241	wheelchair,classification,impairment,cp,paralympic,cerebral,classes,visual,para,basketball

**Fig 3:**
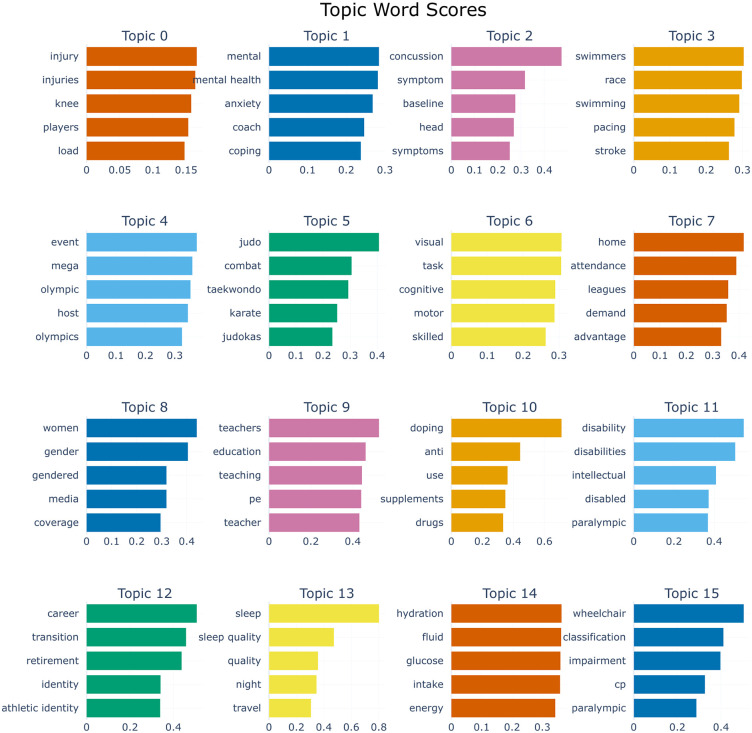
Representative keywords and their corresponding weights for selected research topics.

To visualize the characterization of each topic, BERTopic concatenates all documents within a specific cluster into a single “global document.” By calculating the term frequency within this consolidated text, it generates a cluster-level bag-of-words representation to derive word weights. Fig 3 illustrates the top representative keywords and their corresponding probability weights for selected research topics in competitive sports.Compared to traditional topic modeling approaches, BERTopic demonstrates superior unsupervised semantic extraction capabilities. As evidenced in the figure, the top-ranked keywords for each cluster provide a coherent and self-explanatory description of the underlying subject matter.For instance, Topic 0 (Sports Injury and Load Management), Topic 4 (Mega-event Management), and Topic 6 (Cognitive Skills and Motor Control) all exhibit clear semantic boundaries directly pertaining to athletic participation and governance. Furthermore, the magnitude of the keyword weights explicitly signifies the thematic core of each cluster. Distinctive terms such as “mental” in Topic 1, “concussion” in Topic 2, and “disability” in Topic 11 serve as definitive anchors for their respective research domains.

### 4.2. Analysis of inter-topic relationships

To further elucidate the intrinsic structural relationships among the identified topics, we conducted an empirical exploration using a cosine similarity matrix. This matrix is organized based on hierarchical clustering, ensuring that semantically related topics are grouped adjacently.

**Fig 4:**
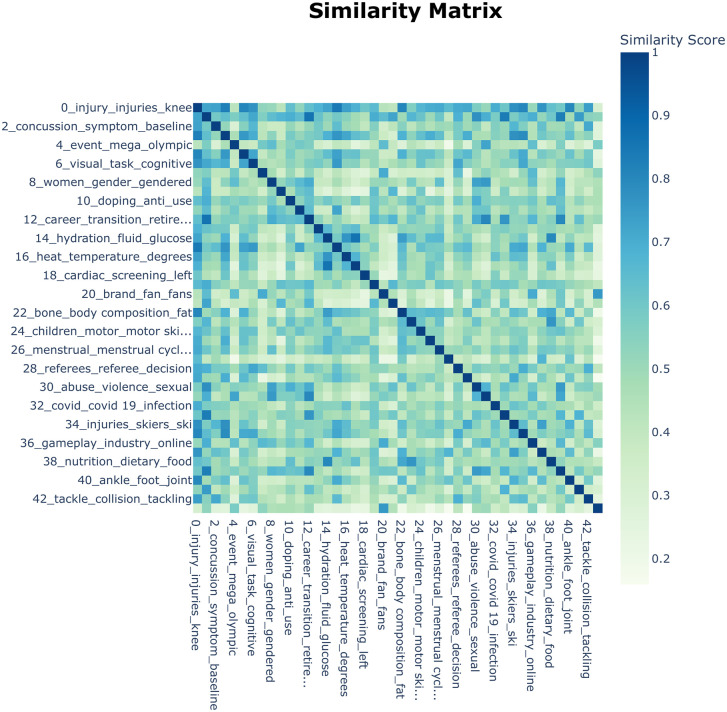
Cosine similarity matrix of research topics in competitive sports.

In this analytical framework, a higher similarity coefficient between two topics signifies a closer semantic proximity and a stronger logical correlation, which manifests as distinct clusters of aggregation within the matrix. As depicted in Fig 4, the visualization employs a color gradient to represent similarity strength: darker tiles correspond to high semantic similarity (approaching a coefficient of 1.0), whereas lighter tiles denote semantic divergence or low correlation.

Overall, the similarity matrix exhibits a well-balanced structural characteristic. The predominance of lighter tiles in the off-diagonal regions indicates that the topics generated by BERTopic represent independent and clearly defined sub-fields. This confirms that the model has successfully disentangled distinct research themes, effectively minimizing semantic redundancy.Specifically, we observed several dense semantic clusters:The Injury-Specific Nexus: Topic 0 (Sports Injury and Load Management) shows high similarity coefficients with Topic 40 (Ankle Injury and Recovery, 0.80) and Topic 42 (Rugby Performance Analysis, 0.74). These high correlations suggest the formation of a dense research network linking general injury mechanisms to specific sports and anatomical joints.The Female Athlete Discourse: A significant correlation (0.64) exists between Topic 8 (Gender Equality and Media) and Topic 26 (Female Athlete Health). This intersection highlights a growing scholarly focus on female athletes that integrates physiological responses with social narratives, distinguishing this discourse from general sports physiology.The Physiological Homeostasis Cluster: Topic 14 (Energy Metabolism), Topic 16 (Heat Adaptation), and Topic 38 (Sports Nutrition) exhibit strong inter-topic similarities (ranging from 0.76 to 0.80). This convergence occurs because these themes all fundamentally pivot around the mechanisms of internal energy balance and recovery.Notably, Topic 20 (Sports Marketing) and Topic 30 (Sports Violence and Protection) display low average similarity scores with other topics. This pattern delineates a clear disciplinary demarcation between sociological research and biological inquiries. Nevertheless, BERTopic confirms that both dimensions remain integral, non-redundant pillars of the competitive sports knowledge system.

**Fig 5:**
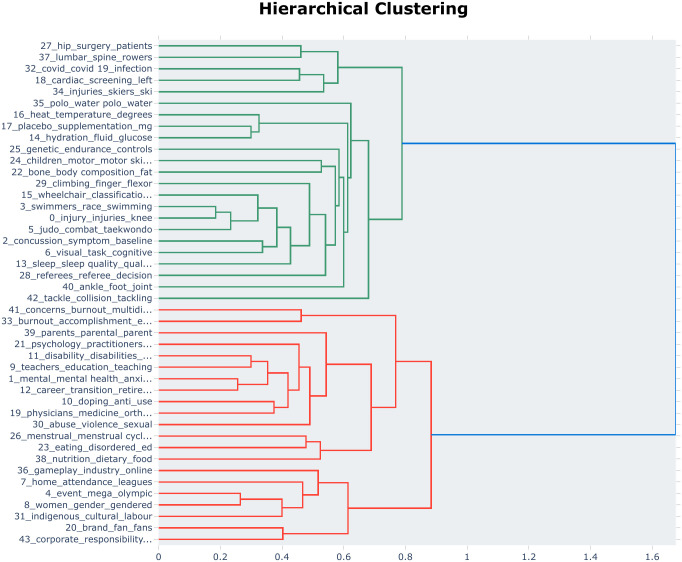
Hierarchical clustering dendrogram of research topics in competitive sports.

To further elucidate the core intellectual structure of the field, we performed hierarchical clustering (as illustrated in Fig 5). When synthesized with the similarity matrix, the results reveal that international competitive sports research is predominantly governed by a dual-paradigm structure: the Biomedical Paradigm and the Social Science Paradigm.The Biomedical Paradigm (Green Cluster): The green cluster aggregates themes related to sports biology, physiology, and anatomy. It encompasses distinct sub-domains such as:Clinical Pathology: e.g., Topic 37 (Rowing Injuries) and Topic 18 (Sports Cardiology and Sudden Death Screening);Endocrinology: e.g., Topic 16 (Heat Adaptation) and Topic 14 (Energy Metabolism);Biomechanics: e.g., Topic 22 (Body Composition Analysis).The Social Science Paradigm (Red Cluster): The red cluster integrates research on cognitive psychology, sociocultural dynamics, and sports marketing. Key components include:Psychology: e.g., Topic 1 (Mental Health) and Topic 33 (Burnout);Sociology: e.g., Topic 8 (Gender Equality and Media) and Topic 30 (Sports Violence and Protection);Management: e.g., Topic 4 (Mega-event Management) and Topic 20 (Sports Marketing).Uncovering Latent Relationships: The hierarchical structure also exposes significant latent associations between seemingly distinct topics:In the Biomedical Cluster: The grouping of Topic 6 (Cognitive Skills and Motor Control), Topic 2 (Concussion/TBI), and Topic 0 (Injury Management) into a single hierarchy implies a theoretical consensus that cognitive function training is inextricably linked to neuro-trauma mechanisms and recovery protocols [38].In the Social Science Cluster: A critical linkage is observed between Topic 26 (Female Athlete Health), Topic 23 (Eating Disorders), and Topic 30 (Sports Violence/Protection). This configuration underscores that for female and marginalized athletes, welfare rights (beyond mere performance) remain insufficiently protected. It highlights an emerging social imperative: safeguarding the well-being of underrepresented groups has evolved from a niche concern into a broader societal issue within the sporting community [39].

**Fig 6:**
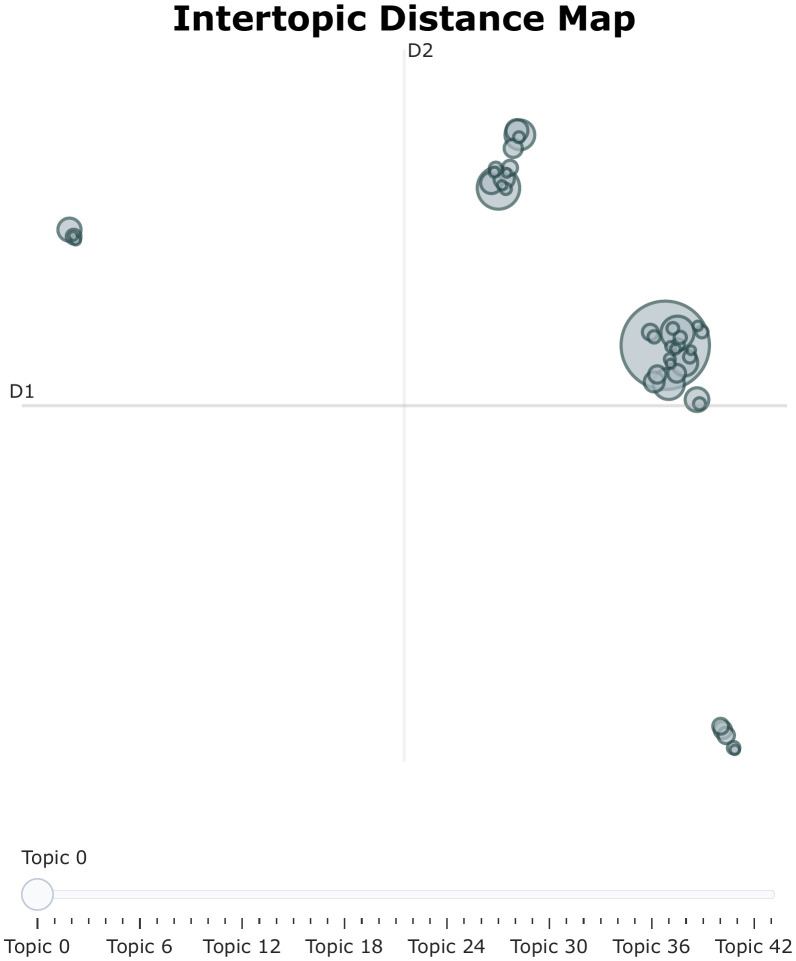
Intertopic distance map visualizing the semantic relationships between competitive sports research topics.

Fig 6 spatially corroborates the dichotomy observed in the hierarchical clustering, further elucidating the relative dominance of different research frontiers. The formation of clear, separated “islands” of bubble clusters in distinct quadrants confirms the discriminative validity of the BERTopic model.Biomedical Dominance (Right Quadrant): The large cluster on the right is anchored by Topic 0 (Sports Injury and Load Management), visually demonstrating its preponderance as the central pillar of competitive sports research. Surrounding this core are satellite themes strictly related to sports medicine, such as Topic 2 (Concussion/TBI) and Topic 18 (Cardiology).Psycho-Social Distinction (Upper Quadrant): In contrast, the upper cluster consists of smaller but tightly packed bubbles, comprising Topic 1 (Mental Health), Topic 8 (Gender/Media), and Topic 11 (Adaptive Sports). These represent the domains of psychology and sociology. Their distinct separation from the biomedical cluster along the Dimension 2 axis suggests that while both are integral to sports science, they operate under fundamentally divergent theoretical frameworks.Emerging and Peripheral Themes: The bubbles in the leftmost and bottom-right quadrants (e.g., Topic 7 Sports Economics, Topic 20 Marketing, Topic 43 CSR) exhibit minimal semantic overlap with the core clusters. This spatial positioning indicates that these are distinct, emerging sub-fields that have established their own representative identities.By integrating the findings from the Similarity Matrix, Hierarchical Clustering, and the Intertopic Distance Map, we have established a unified chain of evidence from point-to-point correlation to geometric spatialization. This triangulation confirms that the field of competitive sports is physiologically defined at its core, yet is an expanding discipline that increasingly incorporates a diverse, multidimensional spectrum of psychological, sociological, and managerial knowledge.

### 4.3. Evolutionary trajectory analysis of research topics

**Fig 7:**
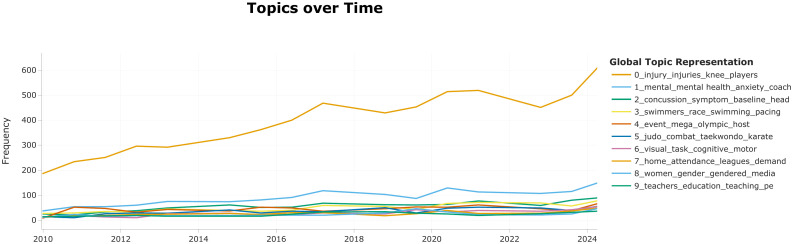
The evolutionary trends of the top 10 research topics in competitive sports (2010–2024).

To intuitively visualize the temporal trends and shifting focus of the field, we employed the Dynamic Topic Modeling (DTM) module within BERTopic. The analysis was conducted with a temporal resolution of one year (time slice = 1 year). To ensure robust and coherent topic representations over time, the following optimization strategies were applied:Temporal The c-TF-IDF matrix at timestamp t-1 was averaged with that of timestamp. This step refines the evolutionary representation by creating a smoother transition between consecutive years.The local c-TF-IDF was further averaged with the global c-TF-IDF matrix (computed across the entire timeframe). This ensures that the dynamic variations remain semantically anchored to the overall topic definitions.Fig 7 illustrates the dynamic evolution of the top 10 identified topics from 2010 to 2024. The temporal distribution reveals a significant surge in publication volume and a marked diversification of research themes. Based on the fluctuations in topic frequency and critical inflection points, the evolutionary trajectory can be categorized into three distinct phases.

T1: The Foundational Incubation Period (2010–2014).During this nascent stage, competitive sports research was characterized by a distinct thematic homogeneity.At the macro level, Topic 0 (Sports Injury and Load Management) established an unassailable dominant position through a sustained, linear growth trajectory, significantly outperforming other themes in frequency. This data pattern signifies that physiological injury prevention and physical rehabilitation constituted the epistemological bedrock of the field's early knowledge system. It further confirms that the academic community's primary focus during this inception phase was heavily concentrated on the biomedical dimensions of sport.At the micro level, while topics such as Topic 3 (Swimming Performance) and Topic 6 (Cognitive Skills and Motor Control) exhibited lower absolute frequencies, they demonstrated remarkable temporal stability. This reflects the robustness of early research paradigms in sports biomechanics and motor control. Scholars prioritized the investigation of determinants of athletic success and cognitive motor tasks, aiming to optimize competitive performance through rigorous measurement.Methodologically, this period was predominantly governed by positivism and experimental paradigms, focusing on quantifiable physiological and biomechanical variables.

T2:The Rapid Expansion Period (2015–2019).During this phase, while Topic 0 (Sports Injury and Load Management) maintained its upward momentum, the dynamics of other themes began to reflect distinct epochal characteristics driven by external events and emerging medical consensus.Event-Driven Management Shift: The fluctuation in Topic 4 (Mega-event Management), particularly the surge aligning with the 2016 Rio Olympic cycle, demonstrates the field's rapid responsiveness to global sporting calendars. This signals a macroscopic shift in attention from the individual athlete to the systematic governance and management of mega-events.The “Cognitive Shift” and Sports Neurology: Concurrently, a profound “cognitive shift” occurred at the micro-level, evidenced by the significant ascent of Topic 2 (Concussion and Traumatic Brain Injury). This trend aligns with heightened global scrutiny regarding Chronic Traumatic Encephalopathy (CTE) and the optimization of concussion protocols. Parallel to this, the increased publication volume in Topic 5 (Combat Sports and Martial Arts) reflects the intensified investigation of head trauma within high-contact disciplines. Collectively, these developments mark a pivotal disciplinary transition during this period: a pivot from pure biomechanics toward sports neurology, prioritizing the long-term neurological welfare of athletes.

T3:The Multidimensional Transformation Period (2020–2024).In recent years, the landscape of competitive sports research has been characterized by heightened volatility and thematic diversification, marking a decisive paradigm shift toward the comprehensive Bio-Psycho-Social Model.While Topic 0 (Sports Injury and Load Management) remains the most prevalent theme, its relative hegemony is increasingly contested by a surge in non-physiological research themes. These fluctuations are inextricably linked to exogenous shocks, most notably the COVID-19 pandemic.Micro-Level Narrative Expansion: The research narrative has expanded beyond the purely somatic dimension to encompass broader psychological and sociological spheres:Mental Resilience: Recognized as a critical determinant of individual agency and athletic performance, mental health has garnered intense scrutiny. This is evidenced by the consistent year-on-year ascent of Topic 1 (Sports Mental Health).Pandemic Catalysts: The suspension of sports leagues and the physical isolation of athletes during the pandemic served as a catalyst, accelerating scholarly attention toward Topic 1 and Topic 7 (Sports Economics), as the industry grappled with existential financial and psychological crises.Social Embeddedness: Furthermore, acknowledging the deep interconnectedness between competitive sports and broader societal dynamics, the prominence of Topic 8 (Gender Equality and Media) highlights a growing academic commitment to social justice and gender equity within sports media representation [40,41]. This confirms that the field is moving towards a more holistic understanding of athletes as socially embedded individuals.

### 4.4. Prediction methodology for future research trends

As shown in Table 2, to rigorously quantify the evolution of research interests within the competitive sports domain, we performed a temporal trend analysis using Ordinary Least Squares (OLS) linear regression on the annual publication frequency of each topic over the last 15 years (2010–2024). The regression results, including slope coefficients, statistical significance, and regression diagnostics, are summarized in Table 2. Addressing the potential for false positives arising from multiple hypothesis testing, we applied the Benjamini-Hochberg procedure to adjust the p-values, thereby controlling the False Discovery Rate (FDR). Furthermore, to ensure the validity of the time-series models, we conducted the Durbin-Watson (DW) test for autocorrelation. The majority of specific research topics (e.g., Topics 1, 2, 3) exhibited DW statistics close to 2.0 (range: 1.8–2.2), indicating independent residuals and a robust fit for the linear model. Broader topics, such as Topic 0 (Injury, knee, players), showed mild positive autocorrelation (DW ≈ 1.3), reflecting the “research inertia” or cumulative advantage characteristic of established, high-volume fields; however, their growth trends remained statistically highly significant (p_adj_ < 0.001).

**Table 2:** Quantitative statistics and regression analysis of research topics in competitive sports (2010–2024).

Topic	N	Trend Slope(β)	P-value	Adjusted *P*-value (FDR)	Durbin-Watson
0	5685	23.21	< 0.001	< 0.001	1.27
1	1298	5.59	< 0.001	< 0.001	1.94
2	800	3.53	< 0.001	< 0.001	1.9
3	705	2.91	< 0.001	< 0.001	2.04
4	651	1.25	0.094	0.115	1.85
5	514	2.29	< 0.001	0.001	2.35
6	398	2.11	< 0.001	< 0.001	2.62
7	382	1.17	0.002	0.005	2.2
8	343	0.97	0.041	0.054	1.27
9	334	1.11	0.003	0.008	0.89
10	303	1.12	< 0.001	< 0.001	1.96
11	287	1.54	< 0.001	< 0.001	2.4
12	233	1.28	< 0.001	0.003	2.72
13	230	0.84	0.007	0.013	1.43
14	211	1.16	< 0.001	0.002	1.11
15	214	1.21	0.001	0.004	2.5
16	190	0.89	< 0.001	0.003	2.53
17	180	0.87	0.002	0.006	2.88
18	184	0.82	0.004	0.009	3.01
19	160	0.74	0.005	0.009	2.14
20	157	0.68	0.022	0.033	2.03
21	129	0.41	0.046	0.059	1.77
22	119	0.09	0.627	0.656	1.62
23	116	0.59	0.009	0.014	2.24
24	118	0.41	0.029	0.04	3.04
25	117	0.58	0.004	0.008	1.1
26	111	0.64	0.003	0.007	1.73
27	114	0.64	0.004	0.008	2.13
28	100	0.73	0.001	0.004	1.28
29	100	0.49	0.008	0.014	2.71
30	83	0.12	0.298	0.335	2.33
31	88	0.23	0.131	0.155	1.7
32	88	0.47	0.008	0.014	1.88
33	74	0.51	0.003	0.008	2.02
34	75	0.36	0.023	0.034	2.04
35	73	0.11	0.513	0.563	1.9
36	64	0.33	0.007	0.013	2.41
37	65	0.2	0.15	0.173	1.84
38	62	0.26	0.077	0.096	1.52
39	67	0.29	0.021	0.033	2.68
40	61	0.06	0.767	0.785	1.12
41	58	0.01	0.933	0.933	2.27
42	55	0.07	0.539	0.577	1.97
43	53	0.22	0.028	0.04	1.54

**Note:** *N:*The document counts in Table 1 represent the total number of articles identified per topic. In contrast, Table 2 includes only those articles with valid publication years falling within the analyzed time window (2010–2024) for trend analysis. Consequently, the totals in Table 2 are slightly lower than those in Table 1 due to the exclusion of undated records or those outside this period. *β*: Slope of the linear regression trend; positive values indicate increasing annual publication volume. *FDR:* False Discovery Rate adjusted *P*-values (Benjamini-Hochberg correction). *Durbin-Watson*: Statistic ranges from 0 to 4. Values near 2.0 indicate independent residuals; values between 1.0–3.0 are generally considered acceptable for bibliometric trend analysis.

Even after rigorous FDR correction, the analysis confirmed significant growth trends for several key thematic clusters. Topic 0 demonstrated the steepest growth trajectory (β = 23.21, p_adj_ < 0.001), solidifying its status as the central pillar of competitive sports research. Notably, Topic 1 and Topic 2 emerged as rapidly expanding “hot” topics (β = 5.59 and 3.53, respectively; p_adj_ < 0.001), reflecting the paradigm shift towards athlete well-being and neurological safety in recent decades. Other statistically significant growth areas included Topic 3 and Topic 5, highlighting the continuous diversification of the field.

## 5. Discussion

By leveraging the BERTopic modeling framework and dynamic trend analysis, this study reconstructs the evolutionary trajectory of knowledge within competitive sports research from large-scale unstructured textual data.Transcending traditional descriptive bibliometrics, this research aims to look beyond superficial data patterns to unveil the underlying cognitive logic and epistemic drivers that govern the field's development. Synthesizing the statistical findings presented above, the following discussion provides a comprehensive interpretation addressing the four central research questions.

### 5.1. Hierarchical modularity of the disciplinary knowledge structure

In response to RQ1 and RQ2, our findings unveil a knowledge ecosystem characterized by a distinct “Core-Periphery” structure.Topic 0 (Sports Injury and Load Management), as the most voluminous cluster, constitutes the epistemic bedrock of the field. This finding empirically corroborates that while competitive sports research increasingly exhibits multidisciplinary characteristics, its ontological essence remains profoundly anchored in the “Biomedical Model.” Although integration with sociological or managerial perspectives is deepening, the interrogation of human physiological limits and somatic functionality remains the fundamental investigative logic of the discipline.Furthermore, the inter-topic relational structure exhibits clear hierarchical modularity. This signals that competitive sports research has evolved from early fragmented empiricism into a mature disciplinary system defined by stable paradigms and a clear academic division of labor.The spatial semantic distance observed between the core (Topic 0) and peripheral sub-themes (e.g., psychology, tactics) serves a dual function: it highlights existing specialization silos (professional barriers) within the discipline, while simultaneously exposing the latent potential for cross-disciplinary synthesis.

### 5.2. Analysis of the evolutionary trajectory

Addressing the diachronic evolution (RQ3), this study reveals a “Dual-Engine Mechanism” driving the development of competitive sports research: the synergy between Sports Medicine and Social Humanities. Here, we deconstruct the deep-seated catalysts behind this phenomenon.

(1) Technological Empowerment and the “Evergreen” Effect: Topic 0 (Sports Injury and Load Management) exhibits not only the highest volume but also the highest growth coefficient (Coefficient = 23.21, Hot). This defies the conventional “lifecycle decay hypothesis” where older topics fade over time. We attribute this “evergreen” vitality to the continuous iteration of research methodologies. With the integration of wearable sensors, computer vision, and AI algorithms, traditional “injury prevention” has been reconstructed into “precision load monitoring and injury recovery.” This co-evolution of technology and theory allows classic themes to be constantly reinvigorated, demonstrating a distinct scientific characteristic of technological empowerment [42]
(2) Paradigm Shift: From “Instrumental Rationality” to “Value Rationality”: The robust emergence of Topic 1 (Sports Mental Health; Coefficient = 5.59, Hot) marks a cognitive revolution within the field. Historically, competitive sports research has been dominated by “Instrumental Rationality,” targeting “athletic performance” as the ultimate objective. However, the explosive growth of Topic 1 signals that the academic community is re-examining the subjectivity of the athlete as a “whole person.” This trajectory—shifting from the singular pursuit of “Faster, Higher, Stronger” to a focus on “Resilience, Burnout, and Well-being”—reflects a profound epistemological correction from Mechanical Reductionism to Humanism. This is not merely a rotation of hot topics; it is an isomorphic projection of broader shifts in sports ethics and sociocultural values onto the scientific landscape [43].
(3) Dynamic Equilibrium in Institutionalized Fields:In contrast to other “Hot” topics, Topic 4 (Mega-event Management) displays a “Neutral” status (p > 0.05). This non-significant linear growth confirms that research in this domain is often driven by pulsatile external events (e.g., Olympic or World Cup cycles). A deeper underlying reason is the “Theoretical Saturation Effect”: macro-management theories (e.g., event legacy, governance) are relatively mature. Contemporary research largely consists of incremental refinements within existing frameworks rather than disruptive innovations. To break this bottleneck, future inquiries may need to pivot toward emerging angles such as digital transformation to reignite growth momentum [44].

### 5.3. Future trends: Multidisciplinary integration and systemic evolution

Based on the evolutionary logic delineated above and addressing RQ4, we postulate that future competitive sports research will evolve toward a “Complex Systems Paradigm.” This evolution will manifest across three distinct dimensions.

Thematic Dimension: Deep Synthesis of the Mind-Body Dualism The parallel high-growth trajectories of Topic 1 (Mental Health) and Topic 0 (Injury/Load) foreshadow that the next frontier lies at their intersection. Future research will be dedicated to constructing “Psycho-Biological Coupling Models.” For instance, investigating the neuromuscular mechanisms through which cognitive fatigue precipitates physical injury will be a critical breakthrough point for dismantling traditional disciplinary silos [45].

Methodological Dimension: The Leap to Computational Predictive Science The infiltration of technological elements within Topic 0 signals a qualitative metamorphosis in methodology. We are witnessing a transition from “Descriptive Analytics” to “Computational Predictive Science.” Future inquiries will leverage multimodal data fusion—integrating video streams, physiological signals, and electronic health records—to achieve real-time forecasting of athletic performance and injury risks. This will drive a fundamental transformation of the field from an empirical science to a data-driven computational science [46].

Practical Dimension: Contextual Reconstruction of Sports Management In the realm of management, a trend of “Contextual Reconstruction” is emerging. The plateauing interest in Topic 4 (Mega-event Management) suggests that its future potential relies not on existing frameworks, but on adapting to new contexts. With the meteoric rise of Esports and Virtual Sports [47,48], management research will pivot from traditional organizational efficiency toward Social Responsibility (CSR), Green Governance, and Digital Experience. This interdisciplinary infusion will revitalize this traditional domain, initiating a new lifecycle of academic relevance.

## 6. Conclusion

### 6.1. Summary of findings

This study successfully achieved a semantic reconstruction of unstructured textual data in the field of competitive sports by employing the BERTopic modeling framework.Our investigation confirms that the discipline has evolved into a complex knowledge system structured around three distinct pillars: physiologically grounded foundations, psychologically driven research frontiers, and managerially modulated latent themes.Empirical evidence demonstrates a high degree of internal cohesion within the field. Crucially, the evolutionary logic of competitive sports research is undergoing a fundamental transition: shifting from a monolithic performance-oriented focus toward a dual paradigm that synergizes technological innovation with humanistic values.

### 6.2. Theoretical and methodological contributions

Methodologically, this study transcends the limitations of traditional co-word analysis, which often exhibits an excessive reliance on high-frequency terms.We successfully demonstrate the superiority of Transformer-based deep learning models in processing complex scientific texts. Specifically, the model demonstrated the capacity to accurately capture the phenomenon of “semantic saturation” in mature topics such as “Mega-event Management.” This provides a novel analytical paradigm for future sports scholarship: shifting the focus from explicit “quantitative growth in publications” to implicit “qualitative semantic dissection.”

### 6.3. Practical implications

Based on the characterized evolution of research topics, we propose the following recommendations for practice and policy:Funding Strategies: In light of the explosive growth of “Sports Mental Health,” funding agencies and academic institutions should establish dedicated grants to incentivize cross-boundary “Bio-Psycho” research. It is imperative to move beyond siloed stagnation and redundant intra-disciplinary competition (often referred to as “involution”), fostering instead a collaborative ecosystem that bridges the physiological and psychological divides.Coaching and Management: Practitioners, including coaches and sports administrators, must recognize that data-driven “Load Management” (Topic 0) has evolved from a scientific concept into an industry imperative. However, sustainable competitive advantage in the future will not derive solely from data collection. Rather, it will depend on the ability to synergize quantitative physiological data with qualitative psychological well-being (Topic 1). This integration is essential for constructing a holistic development framework that nurtures the “whole athlete.”

### 6.4. Limitations and future prospects

While this study offers a comprehensive macro-perspective on the evolution of competitive sports research, several methodological constraints necessitate caution in interpretation. First, the operationalization of “competitive sports” through keyword queries remains an imperfect proxy for the complex reality of sporting practices. Although we rigorously purified the corpus to remove disciplinary noise (e.g., physics or soil science), keyword-based retrieval inherently lacks the semantic nuance of manual qualitative coding. Consequently, our dataset may still encompass studies situated in “peripheral” or non-elite contexts—such as recreational exercise or school-based physical education—where the boundary between “sport” and “play” is linguistically ambiguous. This is an unavoidable trade-off in large-scale bibliometric mapping: we prioritize the breadth of the dataset to capture global trends, accepting a marginal degree of contextual noise that strictly elite-focused manual reviews might exclude. Second, the linguistic scope of this analysis was restricted to English-language publications. This decision, while necessary for the coherence of the BERTopic model, inevitably introduces a geopolitical bias, potentially underrepresenting significant contributions from non-Anglophone sporting powerhouses (e.g., published in Chinese, Russian, or Spanish journals). The trends observed here should be viewed as a reflection of the internationalized academic discourse rather than the totality of global knowledge production.

Future research should aim to transcend these boundaries by integrating advanced Natural Language Processing (NLP) techniques. Specifically, subsequent studies could employ Large Language Models (LLMs) to perform context-aware filtering, distinguishing between “competitive” and “recreational” contexts with greater precision than Boolean operators. Furthermore, adopting a mixed-methods approach—combining computational topic modeling with expert-driven qualitative meta-synthesis—would provide a richer, more granular understanding of how theoretical paradigms in competitive sports are shifting at the micro-level.

## Supporting information

S1 Data Raw bibliographic dataset. This file contains the original metadata records (including abstracts and years) retrieved for the study.
(XLSX)

S2 Code Python script for BERTopic modeling. The script used for document preprocessing, topic generation, and evolution analysis.
(IPYNB)

S3 Table Full list of topic. A detailed table showing for each identified topic.
(XLSX)

S4 Table Full list of topic keywords. A detailed table showing the keywords for each identified topic.
(XLSX)
